# Xenobiotic exposure may confound traditional stress biomarkers: evaluating perfluoropentanoic acid and cadmium effects on largemouth bass (*Micropterus nigricans*) leukocyte profiles

**DOI:** 10.1093/conphys/coag077

**Published:** 2026-10-06

**Authors:** Anthony Ryan Johnson, Tyler Steven Coleman, Jasmine B Nasser, Maggie McGreal, Kyle R Piller, Christopher M Murray

**Affiliations:** Center for Environmental Research, Department of Biological Sciences, Southeastern Louisiana University, 808 North Pine Street, Hammond, LA 70401, USA; Center for Environmental Research, Department of Biological Sciences, Southeastern Louisiana University, 808 North Pine Street, Hammond, LA 70401, USA; Center for Environmental Research, Department of Biological Sciences, Southeastern Louisiana University, 808 North Pine Street, Hammond, LA 70401, USA; Center for Environmental Research, Department of Biological Sciences, Southeastern Louisiana University, 808 North Pine Street, Hammond, LA 70401, USA; Center for Environmental Research, Department of Biological Sciences, Southeastern Louisiana University, 808 North Pine Street, Hammond, LA 70401, USA; Center for Environmental Research, Department of Biological Sciences, Southeastern Louisiana University, 808 North Pine Street, Hammond, LA 70401, USA

**Keywords:** Cadmium, largemouth bass, leukocyte profiles, per- and polyfluoroalkyl substances, perfluoropentanoic acid, stress

## Abstract

Understanding how contaminants influence physiological stress is vital for conserving aquatic organisms in increasingly altered environments. We examined whether exposure to cadmium (Cd) or perfluoropentanoic acid (PFPeA)—a short-chain per- and polyfluoroalkyl substance—altered neutrophil-to-lymphocyte ratios (NLRs) in largemouth bass (Micropterus nigricans) under controlled laboratory conditions. Using NLR as a leukocyte-based biomarker associated with physiological stress responses, we compared five groups (control, Cd low, Cd high, PFPeA low, PFPeA high) across four sampling periods (baseline, post-treatment, 3 h after an abrupt salinity increase and 7 days later). High PFPeA exposure elevated NLR values within 4 days of treatment relative to the other groups. Following salinity exposure, all groups exhibited acute increases in NLR; however, elevated NLR persisted only in the high-PFPeA group 1 week later. Neither Cd treatment nor low PFPeA exposure altered NLR relative to controls. Our findings suggest that high PFPeA exposure may alter leukocyte dynamics and influence the interpretation of leukocyte-based stress biomarkers in freshwater fishes exposed to additional environmental stressors. More broadly, xenobiotic contaminants may complicate the interpretation of physiological biomarkers used in conservation and management contexts.

## Introduction

Physiological biomarkers are increasingly used in conservation physiology to evaluate how organisms respond to environmental perturbations, including habitat degradation, climate change, salinity shifts, and contaminant exposure (Feder, 1987; Wikelski and Cooke, 2006; Cooke *et al.*, 2014). Metrics such as comparative leukocyte profiles (Murray *et al.*, 2013), endocrine markers (Aerts *et al.*, 2015), and relative performance (Kern and Langerhans, 2019) have been used to evaluate disturbance in aquatic systems. Among these biomarkers, leukocyte profiles—particularly neutrophil-to-lymphocyte ratios (NLRs)—have become widely used indicators of vertebrate stress responses because glucocorticoid-mediated stress responses predictably increase circulating neutrophils while decreasing lymphocyte abundance (Davis *et al.*, 2008; Goessling *et al.*, 2015). Elevated NLR values have been associated with acute and chronic environmental stressors across vertebrate taxa and are commonly used to assess stress physiology in ecological and conservation contexts (Watson *et al.*, 2020; Brosset *et al.*, 2021; Mallett *et al.*, 2024).

Environmental contaminants may complicate the interpretation of leukocyte-based stress biomarkers because many xenobiotics can independently alter endocrine and immune function. Per- and polyfluoroalkyl substances (PFASs) are highly persistent anthropogenic compounds (Croteau *et al.*, 2005; Ali *et al.*, 2019; Schymanski *et al.*, 2023; Edo *et al.*, 2024) widely distributed throughout aquatic ecosystems due to decades of industrial and commercial use (Ritter *et al.*, 2002; Lescord *et al.*, 2015; Gaines, 2023). Although long-chain PFAS compounds have historically received the greatest toxicological attention, short-chain replacement compounds are increasingly detected in aquatic systems (Wang *et al.*, 2013; Walsh *et al.*, 2025) and remain comparatively understudied (Brendel *et al.*, 2018; Guillette *et al.*, 2020). Existing evidence suggests PFAS exposure may disrupt endocrine signalling, alter thyroid function, induce oxidative stress, and modify immune responses in fishes and other vertebrates (Fang *et al.*, 2013; Birgersson *et al.*, 2021; Coperchini *et al.*, 2021; Piva *et al.*, 2022). In fishes specifically, PFAS exposure has been associated with altered immune biomarkers, thyroid disruption, and changes in leukocyte abundance (Guillette *et al.*, 2020; Birgersson *et al.*, 2021). Consequently, contaminant exposure may influence leukocyte dynamics and complicate the interpretation of physiological stress biomarkers in response to additional environmental stressors.

In addition to PFAS compounds, heavy metals (e.g. cadmium [Cd]) are widespread aquatic contaminants (Wu *et al.*, 2016; Kahlon *et al.*, 2018) with well-documented physiological and immunological effects in fishes (Javed and Usmani, 2019; Bera *et al.*, 2022; Shahjahan *et al.*, 2022). Exposure to Cd has been linked to oxidative stress, endocrine disruption, altered osmoregulatory function, and immunoregulation across freshwater taxa (Hontela *et al.*, 1992; Jezierska and Witeska, 2006; Jezierska *et al.*, 2009; Atli and Canli, 2010; Liu *et al.*, 2022). PFAS compounds and Cd may alter physiological stress responses through endocrine (Coperchini *et al.*, 2021, 2024; Nayak *et al.*, 2023), immune (Fang *et al.*, 2013, 2026; Guillette *et al.*, 2020), and nervous (Starnes *et al.*, 2022) system disruption. Therefore, xenobiotic exposure may alter how organisms respond to additional environmental challenges (Barton, 2002).

Salinity fluctuations are particularly relevant secondary stressors in many freshwater systems as saltwater intrusion continues to impact our freshwater resources (Barlow and Reichard, 2010; Cao *et al.*, 2024). Abrupt osmotic changes can activate endocrine stress pathways, disrupt ionoregulation, and alter leukocyte dynamics in fishes (Boeuf and Payan, 2001; Barton, 2002; Wang *et al.*, 2019). Evaluating contaminant-associated changes in leukocyte responses following salinity exposure may improve our understanding of how xenobiotic exposure influences the interpretation of stress biomarkers under ecologically relevant multi-stressor conditions.

Although previous studies have demonstrated that contaminants can alter leukocyte profiles and immune function, relatively few studies have evaluated whether xenobiotic exposure modifies leukocyte-based stress responses following secondary environmental perturbations. Understanding these interactions is important because physiological biomarkers are frequently used to assess organismal condition and environmental stress in conservation, fisheries, and ecological monitoring programs (Cooke *et al.*, 2013). Furthermore, while xenobiotics are released into our freshwater ecosystems at unpredictable rates (Pinheiro *et al.*, 2021; Sharma *et al.*, 2025), there is considerable concern about the management of socioeconomically important fishes in freshwater fisheries.

Largemouth bass (*Micropterus nigricans*; Lacépède, 1802; Lesueur, 1822; Kim *et al.*, 2022) are ecologically and economically valuable freshwater predators widely distributed across North America (Tringali *et al.*, 2015; Taylor *et al.*, 2019) that are commonly used in ecotoxicological and fisheries research (Ludsin and DeVries, 1997; Hanson and Cooke, 2009; Kieffer and Cooke, 2009; Coleman *et al.*, 2024). Since largemouth bass are top-level piscivores that frequently inhabit systems exposed to agricultural runoff, industrial discharge, and saltwater intrusion, they are valuable sentinel organisms for evaluating physiological responses in freshwater ecosystems. Previous studies have demonstrated that largemouth bass exhibit measurable physiological and behavioural responses to environmental disturbance, including contaminant exposure and salinity stress (Sloman and Wilson, 2005; Norris *et al.*, 2010; Yi *et al.*, 2021; Song *et al.*, 2024; Xie *et al.*, 2024). Therefore, largemouth bass are suitable for evaluating how xenobiotic exposure may influence the interpretation of leukocyte-based stress biomarkers following secondary environmental perturbations.

Here, we evaluated whether exposure to cadmium (Cd) and perfluoropentanoic acid (PFPeA; a short-chain PFAS compound commonly used in consumer products) altered leukocyte profiles (NLR; a stress biomarker) in largemouth bass before and after an experimental salinity exposure. Previous studies have evaluated the effects of xenobiotic substances on fishes using stress proxies (e.g. Hontela *et al.*, 1992; Wilson *et al.*, 1994; Baker *et al.*, 2013; Seriani *et al.*, 2015a, 2015b; Corrêa *et al.*, 2017; Correia *et al.*, 2019; Rodgers *et al.*, 2019; Witeska *et al.*, 2023), but none, to our knowledge, have tested interactions between xenobiotics and environmental stressors on biomarker utility. We hypothesized that xenobiotic exposure would both elevate NLR, with stronger responses at higher concentrations, and that NLR would remain elevated following salt exposure (Fig. 1). Understanding these interactions between contaminants and environmental stressors is important for improving freshwater ecosystem management and interpreting stress physiology in natural populations.

**Figure 1:**
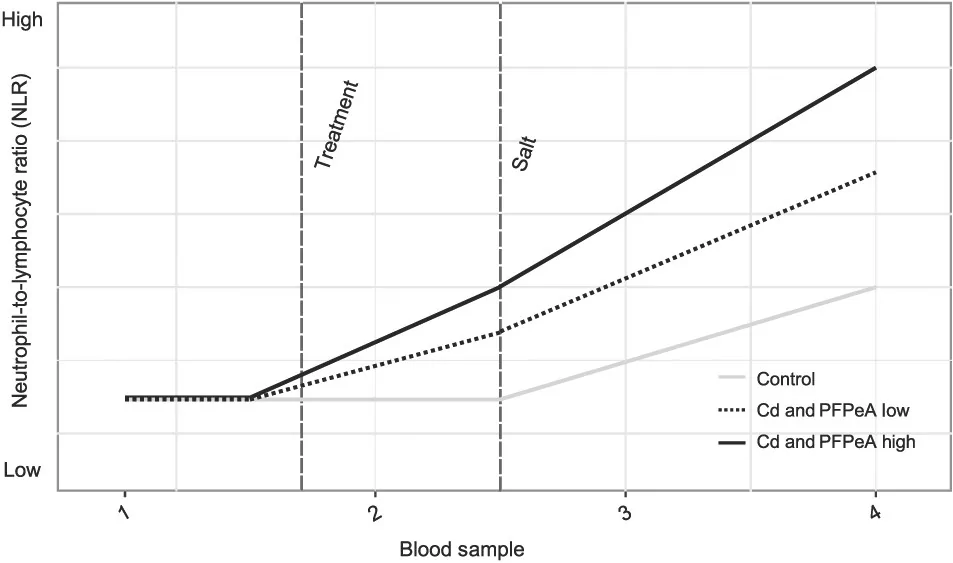
Hypothesized relationships among largemouth bass NLRs, low and high levels of cadmium (Cd) and perfluoropentanoic acid (PFPeA), and an environmental stressor (salinity increase). We anticipated that exposure to Cd or PFPeA, indicated by the dashed ‘treatment’ line between blood samples 1 and 2, would lead to elevated NLR values, particularly in groups receiving higher doses of each toxin. Furthermore, we expected the NLR to increase continuously in all fish after exposure to an abruptly introduced environmental stressor, as represented by the dashed ‘salt’ line between blood samples 2 and 3.

## Materials and methods

### Aquaculture tank design

We assembled 15–37.9 l of rectangular tanks across three shelves, with five tanks per row (Fig. 2). We positioned opaque barriers along the elongated sides of each tank and placed a fine-mesh cover on top. Each tank contained 34.6 l of tap water treated with Seachem Prime concentrated conditioner (Seachem Laboratories, Madison, GA, USA) and a 25-mm aerator (multiport air pump; Aquatic Ecosystems Inc., Apopka, FL, USA). We kept the room under constant low-indirect light throughout the study.

**Figure 2:**
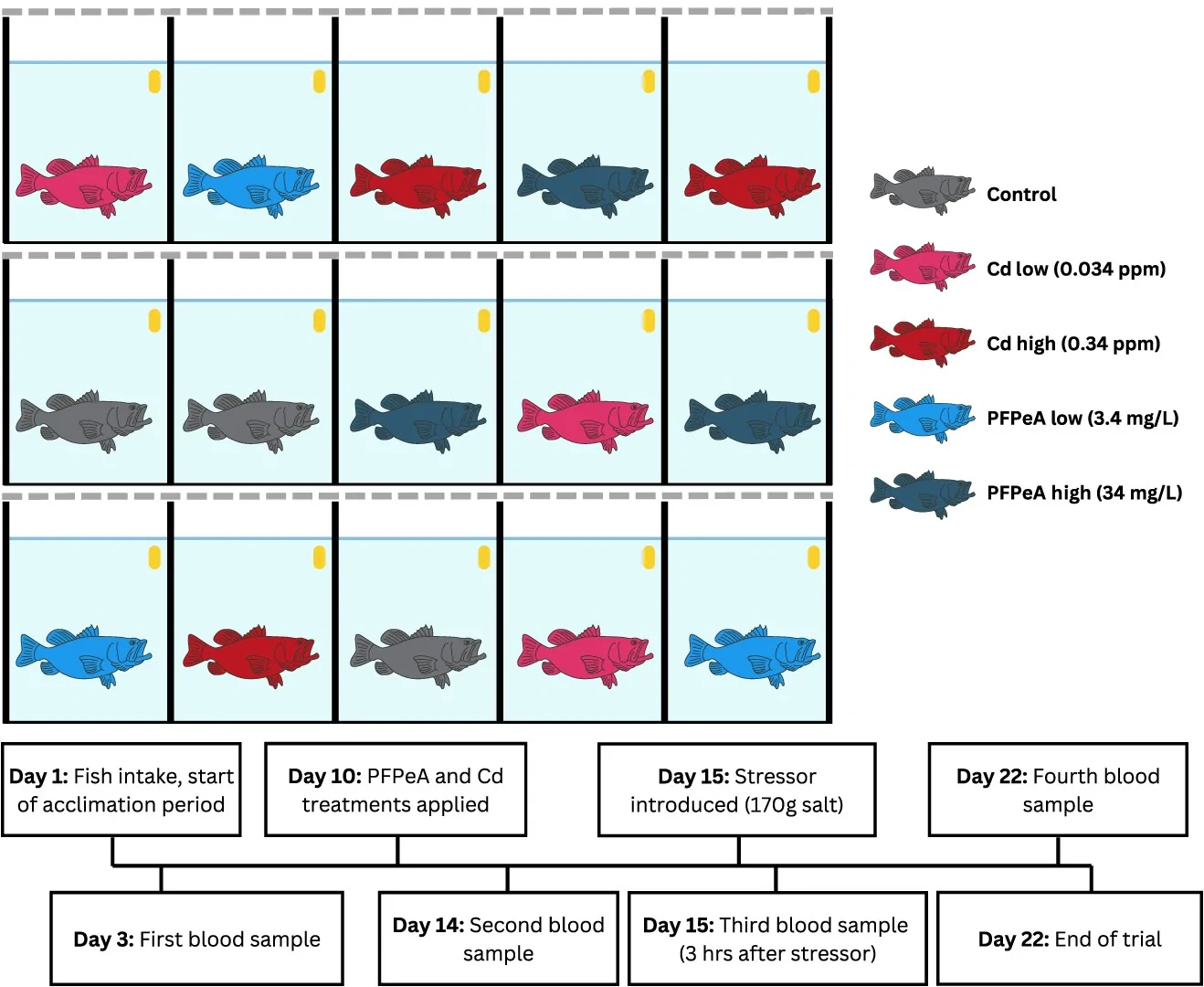
Overview of study design and experimental timeline. We arranged 15 largemouth bass in individual 37.9-l rectangular tanks in three rows, with opaque boundaries along the long sides (indicated by thick solid black bars) and a mesh top (indicated by dashed grey bars). Each tank contained 37.6 l of conditioned tap water (light blue) and was equipped with an aerator (yellow bubbler). We randomized the treatment order across the tanks (five groups, each represented by a different colour). Below the tank design, in connected boxes, we illustrate the chronological order of significant daily events from the start (Day 1) to the conclusion of the trial (Day 22).

Each tank housed a single largemouth bass. Therefore, the individual fish and tank were synonymous experimental units throughout the study. We randomly assigned each tank to one of five groups using a random number generator—control, Cd low (0.034 ppm), Cd high (0.34 ppm), PFPeA low (3.4 mg/l), PFPeA high (34 mg/l)—with three fish per group (Fig. 2).

The PFPeA high concentration (34 mg/l) exceeded PFAS concentrations observed in natural aquatic systems (Pan *et al.*, 2014; Lescord *et al.*, 2015; Mussabek *et al.*, 2023). However, the PFPeA low group (3.4 mg/l) represented concentrations more consistent with reported environmental observations in aquatic systems (Kurwadkar *et al.*, 2022). Similarly, reported Cd concentrations in aquatic systems may range from ~0.2 to 0.4 mg/l (Cossa, 1988; Anzecc, 2000). We used an order-of-magnitude scale to evaluate a range of potential physiological responses under controlled laboratory conditions, including concentrations exceeding environmentally typical levels. Experimental toxicology studies commonly include elevated concentrations when toxicological thresholds and biomarker responses remain poorly understood (Everds *et al.*, 2013; Lewis *et al.*, 2024).

We used 15 individuals (three fish per group) to balance replication with available space, animal-care considerations, and the need for repeated blood sampling. Throughout the study, we monitored the fish daily and did not feed the bass to minimize variation in physiological responses associated with differential feeding activity and digestion during repeated blood sampling.

### Experimental timeline

Our study lasted 22 days (Fig. 2). On Day 1, the Louisiana Department of Wildlife and Fisheries supplied bass collected through boat electrofishing from holding ponds at the Huey P. Long Fish Hatchery in Lacombe, LA, USA. Louisiana Department of Wildlife and Fisheries employees promptly transported the fish in an aerated livewell to Southeastern Louisiana University in Hammond, LA, USA. Upon arrival, we recorded the total length and body mass of 15 bass and placed each individual into their assigned tank (aquaculture design described in the *Aquaculture tank design* section above; Fig. 2).

We allowed the fish to acclimate for 2 days (Days 1–2). On Day 3, we collected the first blood sample from the caudal vein of each fish within 3 min of handling without anaesthetics—minimizing any effect of newly induced anthropogenic stress (Davis, 2005; Lawrence *et al.*, 2020)—using a 1-ml syringe and a 25-gauge needle, then re-recorded total length and body mass. On Day 8, we completed partial water changes in all tanks using a DEKO Tools water pump (model CSP9028) to maintain water quality. We did not conduct any additional water changes following contaminant introduction.

To examine whether differing levels of Cd or PFPeA altered baseline NLR, we applied low and high concentrations of Cd (0.034 ppm and 0.34 ppm, respectively) and PFPeA (3.4 and 34 mg/l, respectively; Sigma Aldrich, Darmstadt, Germany, CAS No. 2706-90-3, CF_3_(CF_2_)_3_COOH) directly to the water on Day 10, while the control tanks remained undisturbed. We dissolved both compounds directly into tank water without the use of a carrier solvent; therefore, a solvent control was unnecessary. We analytically verified the masses of Cd and PFPeA added to each tank to ensure consistent nominal concentrations across treatments, but we did not analytically confirm the aqueous contaminant concentrations during the study. On Day 14, we collected total length, body mass, and a second blood sample as before.

On Day 15, to evaluate whether contaminant exposure altered leukocyte-based stress responses following salinity increase, we abruptly increased salinity in all tanks from 0 to ~5 ppt using fast-dissolving Instant Ocean Sea Salt (Figs 2 and 3). We gently mixed the tanks after adding salt to ensure consistent salinity conditions across the tanks and verified salinity immediately after the salt dissolved. Three hours after salt introduction, we collected a third blood sample, total length and body mass, as before. On Day 22, 12 days post-treatment and 7 days post-salt, we collected a final blood sample as before and euthanized all bass using MS-222. The Southeastern Louisiana University Institutional Animal Care and Use Committee (IACUC) approved all procedures described above (IACUC approval 0093).

**Figure 3:**
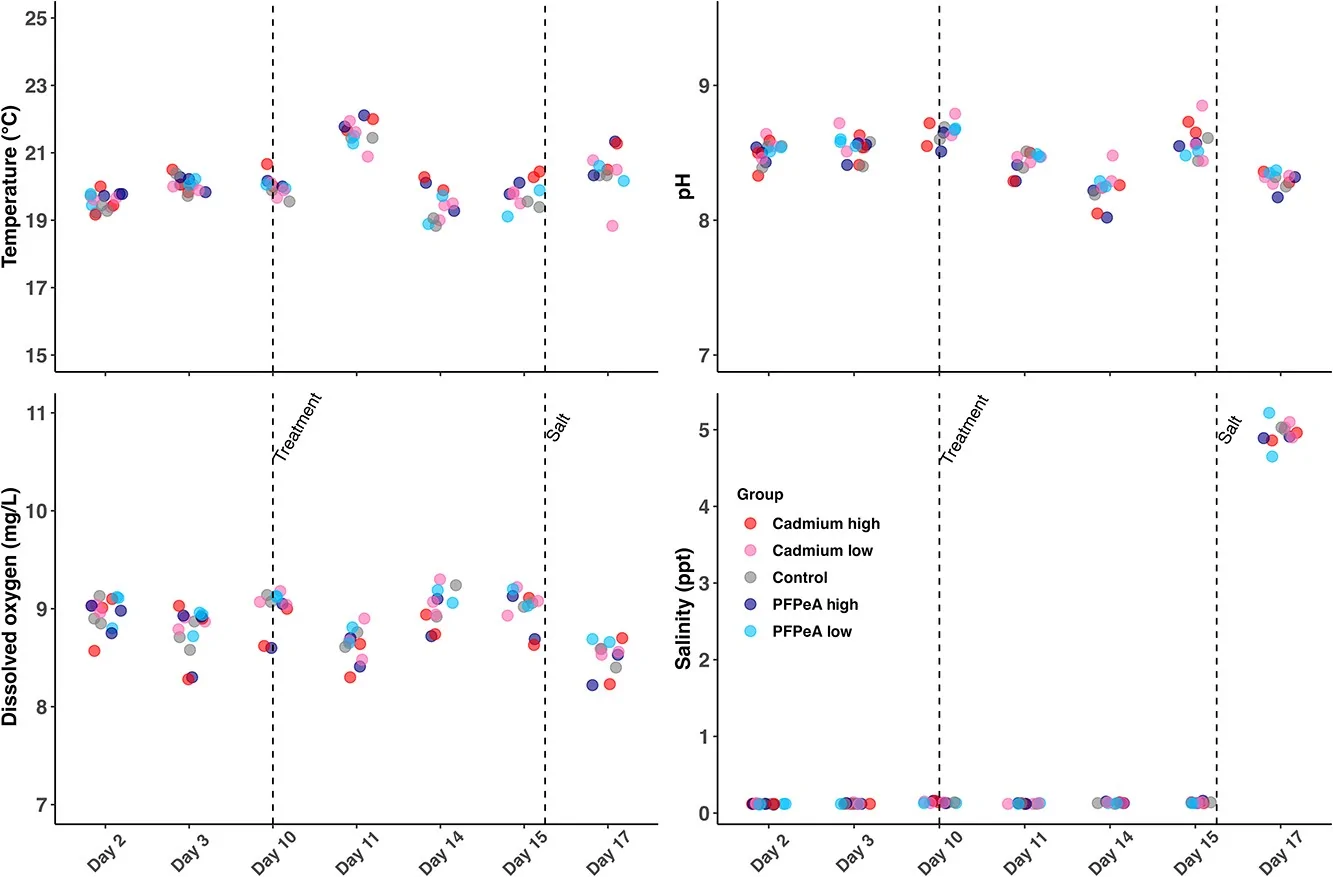
Individual tank water quality data (temperature, pH, dissolved oxygen, and salinity) collected from a YSI Pro DSS on each sampling day (Fig. 2). Colour denotes the treatment group (control and low and high levels of cadmium [Cd] and perfluoropentanoic acid [PFPeA]). The first vertical dashed line indicates the day we added treatments, while the second marks the day we added salt. Note that the reported YSI data on Day 15 are before salt addition.

### Neutrophil-to-lymphocyte ratio

Interest in leukocyte profiles as chronic stress biomarkers has been highlighted in the literature because, unlike glucocorticoids, leukocyte profiles do not attenuate when a stressor persists chronically and are less affected by metabolic fluctuations (Davis *et al.*, 2008; Goessling *et al.*, 2015). Thus, we utilized leukocyte profiles herein to evaluate stress-associated physiological responses.

Immediately after blood collection, we prepared smears using ~5–10 μl of whole blood on standard glass microscope slides (75 × 25 mm; 1 mm thick) with a two-slide wedge method (Houwen, 2002; Murray *et al.*, 2013). After air-drying, we fixed and stained slides with Giemsa–Wright (Fisherbrand, Pittsburgh, PA, USA). We examined smears under a light microscope (40× magnification), identified the first ~100 leukocytes, and calculated the NLR by dividing the neutrophil count by the lymphocyte count. One observer, blind to treatment groups, identified all leukocytes to ensure consistency among samples.

### Water quality measurements

We measured water quality variables—temperature (°C), dissolved oxygen (mg/l), pH, and salinity (ppt)—using a YSI Pro DSS (Yellow Springs Instruments; Xylem Inc., Washington DC, USA) to ensure consistency among tanks. We collected measurements using the YSI on Days 2, 3, 10, 11, 14, and 15 (before and after salinity increase), and again on Day 17 to verify the consistency of the intentional salinity increase. We double rinsed the YSI probe with tap and reverse-osmosis water between tanks to prevent contamination. We used water-quality data to verify consistent environmental conditions across tanks.

### Statistical analyses

We visualized and analysed all data using R within Posit’s RStudio Integrated Development Environment (Posit team, 2025; R Core Team, 2025). For statistical testing, we used an alpha value of 0.05.

Between Days 4 and 7, one fish from the control, Cd high, and PFPeA high groups died. A fish from the PFPeA low group died on Day 14 before we collected the second blood sample. We included all blood and water quality data collected before mortality in subsequent analyses. Table 1 provides sample sizes by group and blood sampling period.

**Table 1:** Number of largemouth bass contributing blood samples within each group—control, cadmium [Cd] low (0.034 ppm), Cd high (0.34 ppm), perfluoropentanoic acid [PFPeA] low (3.4 mg/l), and PFPeA high (34 mg/l)—during each sampling period

Group	Blood 1	Blood 2	Blood 3	Blood 4
Control	3	2	2	2
Cd low	3	3	3	3
Cd high	3	2	2	2
PFPeA low	3	2	2	2
PFPeA high	3	2	2	2

Blood sample 1 was collected before contaminant exposure, blood sample 2 after contaminant exposure, blood sample 3 three hours after salinity exposure, and blood sample 4 seven days after salinity exposure. Mortality reduced sample sizes in several treatment groups following the first sampling period.

First, we qualitatively assessed water quality variables throughout the study (Days 2, 3, 10, 11, 14, 15, 17) to ensure temporal consistency among tanks. We then used the total length and body mass data to calculate each individual’s relative weight (*W*_r_), a commonly used condition metric for freshwater fishes (Neumann and Allen, 2007). We used the regression equation for largemouth bass from Anderson and Neumann (1996) to calculate the standard weight (*W*_s_) required in the relative weight (*W*_r_) equation.

To evaluate whether Cd and PFPeA exposure altered NLRs across sampling periods, we used a generalized linear mixed-effects model (GLMM) fit with the *glmmTMB* package (McGillycuddy *et al.*, 2025). Since NLR values were continuous and strictly positive, we modelled NLR using a Gamma distribution with a log link function. We evaluated distributional fit using the *fitdistrplus* package (Delignette-Muller and Dutang, 2015). Fixed effects included group (control, Cd low, Cd high, PFPeA low, PFPeA high), blood sampling period (blood samples 1–4) and their interaction. We included individual fish identity as a random intercept to account for repeated measurements collected from the same individual across time.

To evaluate group-specific responses across sampling periods, we releveled groups and blood sampling periods to examine targeted model estimates by group and sampling period. Specifically, we evaluated group-associated changes in NLR following contaminant exposure (blood 1 and blood 2), acute salinity exposure (blood 2 and blood 3), and the final post-salinity sampling period (blood 3 and blood 4), including group-specific differences during the final blood sampling period. We assessed model assumptions using residual diagnostics and visually compared the model’s predicted estimates with the observed data distributions using non-parametric bootstrapping.

## Results

### Largemouth bass

On Day 1, bass had a mean total length of 183.87 mm [±15.92 mm; 95% confidence interval (CI)] and a mean *relative weight* of 93.67 (±3.13). By Day 22 (conclusion of the study), mean total length remained unchanged, while mean *relative weight* declined slightly to 87.64 (±4.79). Mean total lengths among groups were similar: Cd low = 164.20 mm (±6.70 mm), Cd high = 204.92 mm (±15.15 mm), PFPeA low = 171.25 (±17.49 mm), PFPeA high = 196.50 mm (±24.35 mm), and control = 184.42 mm (±16.97 mm). We examined 48 blood smears across all groups (12 Cd low, 9 Cd high, 9 PFPeA low, 9 PFPeA high, 9 control). Across all smears, lymphocyte counts ranged from 13 to 70 cells, whereas neutrophil counts ranged from 30 to 86 cells. NLR values ranged from 0.4 to 6.6 across all individuals.

### Water quality

Water temperature, dissolved oxygen and pH remained consistent among tanks throughout the study (Table 2 and Fig. 3). Salinity increased only after we added salt, rising from a mean of 0.13 ppt (±0.0024 ppt; 95% CI) across all tanks before salt intrusion to 4.96 ppt (±0.099 ppt) after salt addition. Water quality values confirmed the experimental salinity increase and indicated stable environmental conditions throughout the study (Table 2 and Fig. 3).

**Table 2:** Water quality summary statistics [minimum (min), mean, maximum (max) and 95% CI] among aquaculture tanks throughout the study (see Fig. 3)

Variable	Min	Mean	Max	95% CI
Temp	18.83	20.10	22.11	±0.16
DO	8.22	8.85	9.30	±0.056
pH	8.02	8.47	8.85	±0.035
Pre-salt	0.11	0.13	0.16	±0.0024
Post-salt	4.65	4.96	5.22	±0.099

The water quality variables included temperature (temp; °C), dissolved oxygen (DO; mg/l), pH, and salinity (salt; ppt), measured using a YSI Pro DSS. Note that ‘pre-salt’ refers to all samples collected before the salinity increase (Days 2, 3, 10, 11, 14, and 15), while ‘post-salt’ denotes the samples from Days 15 and 17, which confirmed the intentional salinity increase. All numeric values are maintained to at least two decimal places while preserving two significant figures.

### Contaminant exposure and salinity effects on NLRs

#### Contaminant exposure (blood samples 1 vs 2)

NLR in the control group did not change between blood samples 1 (Day 3) and 2 (Day 14), representing the pre- and post-toxin periods (*Z*_15,48_ = 0.28; *P* = 0.89; Fig. 4). Neither Cd treatment significantly altered NLR (Cd low: *Z*_15,48_ = 0.24; *P* = 0.81; Cd high: *Z*_15,48_ = 0.30; *P* = 0.32; Fig. 4). PFPeA low showed no effect (*Z*_15,48_ = 0.27; *P* = 0.078; Fig. 4). However, NLR in the PFPeA high group increased by 212% [79–444%; 95% confidence limit (CL)] from blood 1 to blood 2 (*Z*_15,48_ = 0.28; *P* < 0.001; Fig. 4).

**Figure 4:**
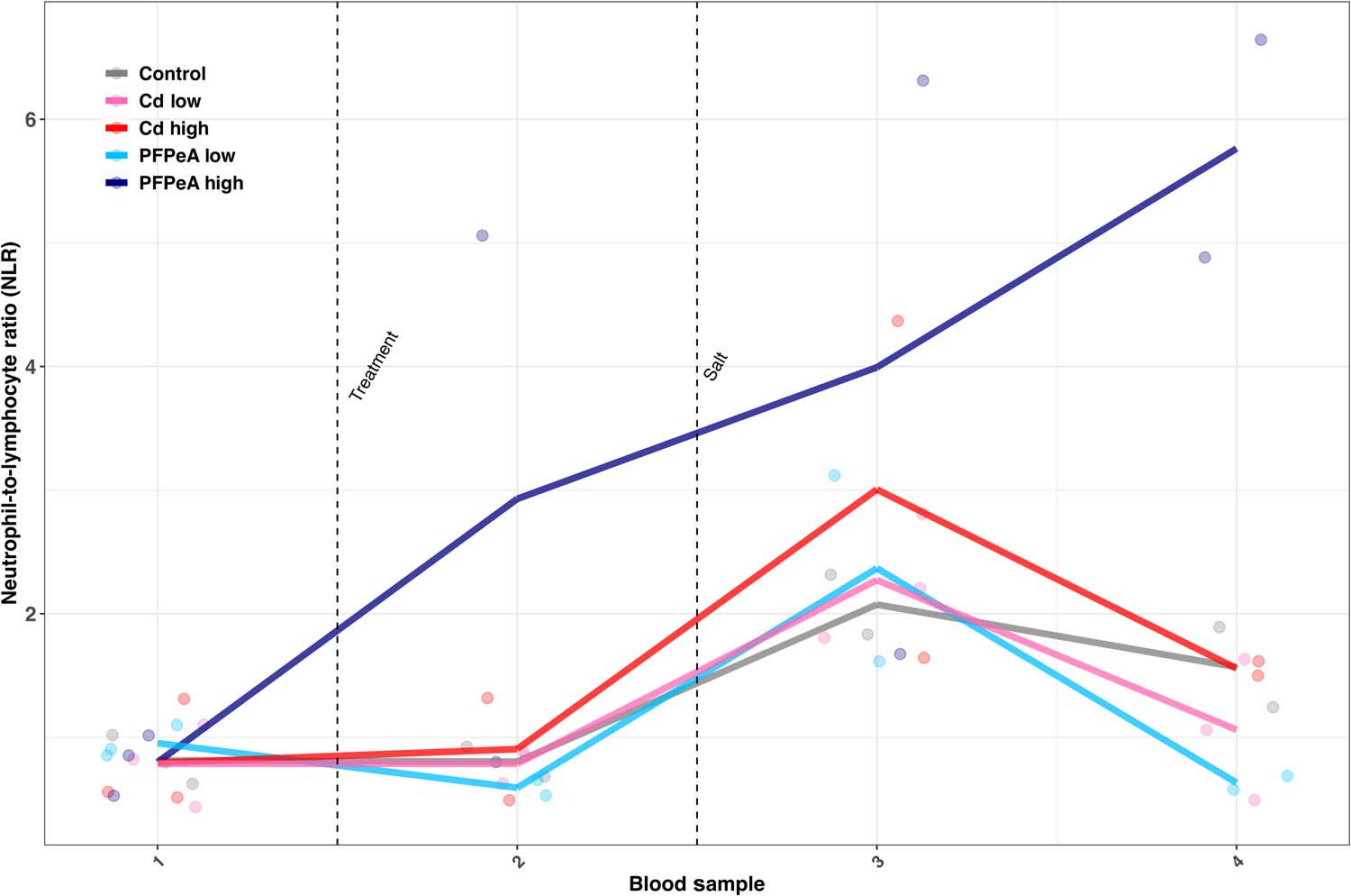
Largemouth bass NLRs from blood smears throughout the study. The five colours represent individual groups: low and high levels of cadmium (Cd) and perfluoropentanoic acid (PFPeA), and the control. Lines dictate the GLMM estimated relationships among the groups and blood samples (slopes can vary due to the group-by-blood-sample interaction). Jittered points represent individual bass NLR at each blood sample. Exposure to Cd or PFPeA is indicated by the dashed ‘treatment’ line between blood samples 1 and 2. Exposure to an environmental stressor is represented by the dashed ‘salt’ line between blood samples 2 and 3.

The PFPeA high bass NLR increased significantly more than all other groups from blood 1 to blood 2: 201% (37–559%; 95% CL), 195% (42–513%), 133% (3–429%), and 407% (134–996%) more than control (*Z*_15,48_ = −2.75; *P* = 0.0059), Cd low (*Z*_15,48_ = −2.90; *P* = 0.0037), Cd high (*Z*_15,48_ = −2.03; *P* = 0.042), and PFPeA low (*Z*_15,48_ = −4.13; *P* < 0.001) groups, respectively (Fig. 4).

#### Acute salinity exposure (blood samples 2 vs 3)

All groups showed a significant increase in NLR following salt introduction (Table 3 and Fig. 4). However, the NLR increases were smaller in the PFPeA high group than in the other groups. Control NLR rose 78% (−21–301%; 95% CL) more than PFPeA high (*Z*_15,48_ = 1.40; *P* = 0.16), while Cd low (*Z*_15,48_ = 1.75; *P* = 0.080) and Cd high (*Z*_15,48_ = 1.95; *P* = 0.051) groups rose 94% (−8–307%) and 127% (−1–418%) more, respectively (Fig. 4). PFPeA low increased 173% (21–515%) more than the PFPeA high group (*Z*_15,48_ = 2.42; *P* = 0.016; Fig. 4).

**Table 3:** The estimated effect sizes of a known environmental stressor (abrupt salinity increase) on largemouth bass NLRs among the five groups (control and low and high levels of cadmium [Cd] and perfluoropentanoic acid [PFPeA]) between blood samples 2 and 3 from the GLMM (Fig. 4)

Group	Effect size	95% CL	*Z*	*P*
**Control**	158%	46, 357	3.25_15,48_	0.0012
**Cd low**	180%	76, 347	4.32_15,48_	<0.001
**Cd high**	228%	81, 494	3.93_15,48_	<0.001
**PFPeA low**	294%	122, 601	4.67_15,48_	<0.001
**PFPeA high**	144%	34, 343	2.91_15,48_	0.0036

The effect size is expressed as a percentage increase from blood 2 to blood 3 [e.g. the control bass NLR increased by 158% (46–357%; 95% CL) from blood 2 to blood 3]. The 95% CLs represent the lower and upper bounds, separated by a comma, such that 95% of repeated studies would contain the true effect size. The *Z* value is reported with the numerator and denominator degrees of freedom. Bolded groups represent statistically significant changes from blood samples 2 to 3.

#### Post-salinity exposure (blood samples 3 vs 4)

By Day 22, NLR declined in all groups except PFPeA high (Table 4 and Fig. 4). NLR in the PFPeA high group continued to exhibit elevated NLR values, and was 271% (75–688%; 95% CL), 223% (42–635%), 526% (176–1319%), and 123% (−2–407%) greater than Cd low (*Z*_15,48_ = 3.42; *P* < 0.001), Cd high (*Z*_15,48_ = 2.80; *P* = 0.0051), PFPeA low (*Z*_15,48_ = 4.40; *P* < 0.001), and the control (*Z*_15,48_ = 1.92; *P* = 0.054), respectively (Fig. 4).

**Table 4:** The estimated effect sizes of xenobiotic substances on largemouth bass 7 days after a known environmental stressor introduction (blood samples 3 to 4) on NLRs among the five groups (control and low and high levels of cadmium [Cd] and perfluoropentanoic acid [PFPeA]) from the GLMM (Fig. 4)

Group	Effect size	95% CL	*Z*	*P*
Control	−25%	−58, 34	−0.97_15,48_	0.33
**Cd low**	−55%	−72, −27	−3.30_15,48_	<0.001
**Cd high**	−48%	−71, −7	−2.22_15,48_	0.026
**PFPeA low**	−73%	−85, −52	−4.50_15,48_	<0.001
PFPeA high	68%	−6, 202	1.75_15,48_	0.081

The effect size is expressed as a percentage change from blood 3 to blood 4 [e.g. the control bass NLR decreased by 25% (−58% to 34%; 95% CL) from blood 3 to blood 4]. The 95% CLs represent the lower and upper bounds, separated by a comma. The *Z* value is reported with the numerator and denominator degrees of freedom. Bolded groups represent statistically significant changes from blood samples 3 to 4.

At the end of the study, NLR remained significantly higher in the PFPeA high bass than in all other treatments (*P* < 0.001 for all comparisons; Fig. 4). NLR values in the control, Cd low, Cd high, and PFPeA low groups did not differ from each other (Fig. 4).

## Discussion

### PFPeA, not Cd, altered leukocyte-based stress

In this study, we evaluated the effects of two concerning and persistent contaminants—cadmium (Cd) and perfluoropentanoic acid (PFPeA)—at different exposure levels on leukocyte-based stress responses in largemouth bass before and after exposure to a known environmental stressor. Our findings are important because little is known about how these contaminants influence physiological biomarkers commonly used in fishes. The control, Cd low, Cd high, and PFPeA low treatments exhibited relatively stable NLR values throughout the study, whereas bass exposed to high PFPeA concentrations exhibited sustained NLR elevation.

We initially hypothesized that Cd and PFPeA would increase NLR, particularly at higher doses, and that all groups would exhibit elevated leukocyte-based stress responses after the salinity increase (Fig. 1). However, only the PFPeA high group showed consistently elevated NLR throughout our study. After salt exposure, NLR in the other four groups declined to near-baseline levels, suggesting that these fish recovered normally from the environmental disturbance, whereas NLR remained elevated in the PFPeA high group.

To date, PFAS exposure has been associated with diverse physiological disruptions with substantial ecological and health consequences (Mallett *et al.*, 2024). For instance, recent reviews have suggested that increased PFAS exposure increases rates of cancer (Steenland and Winquist, 2021), thyroid disruption (Coperchini *et al.*, 2021), and liver impairment (Costello *et al.*, 2022), and it reduces reproductive potential and juvenile fitness in animals and humans (Chambers *et al.*, 2021). Despite these findings, uncertainties remain because research on PFAS exposure is relatively new, often focuses on a limited subset of PFAS compounds, sometimes yields conflicting results, and remains restricted to a few organisms and exposure scenarios. Our results contribute to this growing body of literature by suggesting that PFPeA, a short-chain PFAS compound, may alter leukocyte dynamics associated with physiological stress responses in vertebrates—a directly relevant component of conservation physiology (Cooke *et al.*, 2013).

### Biotic and abiotic consistency

Throughout the study, we took multiple steps to ensure biological and environmental consistency. We intentionally used bass of similar sizes because reducing size variation was important for this experiment, as leukocyte profiles may positively correlate with vertebrate body size (Jaffer *et al.*, 2017; Downs *et al.*, 2020; Naidenko and Alshinetskiy, 2020; Cornelius Ruhs *et al.*, 2021). We restricted food intake during the study, as food consumption can alter metabolic activity, increasing the risk of food-induced changes in leukocyte circulation (Song *et al.*, 2024; Xie *et al.*, 2024). Animals recently introduced to captivity may begin feeding at different times and rates, leading to variability in metabolic activity and immune responses that could confound the effects of contaminant exposure. As expected, we observed a slight decline in relative weight, likely reflecting restricted feeding and confinement, which we deemed acceptable for our study.

Water quality variables remained stable across tanks, confirming consistent environmental conditions. Maintaining consistent abiotic parameters is essential for this type of research, as water conditions can influence the prevalence of various circulating leukocytes in fish species (Corrêa *et al.*, 2017; Shahjahan *et al.*, 2018; Hertika *et al.*, 2022, 2025). By maintaining constant environmental conditions, we reduced the likelihood that observed differences in NLR reflected abiotic variation among tanks.

### Contaminant exposure (blood samples 1 vs 2)

Our study provides new evidence that PFPeA exposure may alter leukocyte-based stress responses in freshwater fishes and further supports the utility of NLR as a physiological biomarker associated with stress responses (Davis *et al.*, 2008; Goessling *et al.*, 2015). After introducing the contaminants, we observed sustained NLR elevation in the PFPeA high group, whereas NLR remained comparatively stable in the Cd low, Cd high, PFPeA low, and control groups. As noted earlier, the high PFPeA and Cd doses exceeded concentrations currently detected in natural aquatic systems. We intentionally incorporated an order-of-magnitude dosing scale approach to evaluate physiological responses across a range of contaminant concentrations under controlled laboratory conditions. Such approaches are common in exploratory toxicology because physiological thresholds and biomarker responses are often poorly understood, especially when investigating understudied compounds, such as short-chain PFASs. These order-of-magnitude approaches enhance our understanding for future research.

Our findings align with previous studies indicating that PFAS exposure can alter physiological processes associated with stress responses in fishes. For instance, Schumann *et al.* (2024) found that freshwater lotic systems with higher PFAS exposure rates were associated with higher plasma cortisol levels. Notably, PFAS compounds’ potential to influence stress-associated physiological processes may pose a significant challenge for fisheries conservation and management, as chronic stress may reduce growth and reproductive rates and increase mortality (Pankhurst and Van Der Kraak, 1997; Schreck, 2010).

Unlike PFPeA exposure, our study suggests that Cd may not induce a stress response in bass. The absence of a Cd effect was unexpected but aligns with previous research on Cd exposure and fish leukocytes, given the duration of our study. For example, the adverse effects of Cd on fishes often emerge with longer exposure durations (Cai *et al.*, 2020; Hu *et al.*, 2021; Liu *et al.*, 2022), and other heavy metals can significantly affect haematological parameters over time (Seriani *et al.*, 2015b). In the freshwater rosy barb (*Puntius conchonius*), Gill and Pant (1985) discovered that circulating lymphocytes increased after Cd exposure, while neutrophils did not, at 0.63 and 0.84 mg/l of Cd exposure over 90 days. These results suggest that Cd-associated changes in leukocyte profiles may be less detectable over our relatively short 12-day exposure window. Nevertheless, the potential impact of Cd on aquatic organism stress should not be dismissed. Cd remains a major heavy-metal toxicant with detrimental effects in aquatic ecosystems, warranting further exploration.

### Acute salinity exposure (blood samples 2 vs 3)

Saltwater intrusion poses a major challenge for coastal communities and ecosystem managers, particularly because it can have additive, synergistic, or antagonistic effects on freshwater, estuarine, and coastal aquatic organisms when combined with other stressors (Barlow and Reichard, 2010; Velasco *et al.*, 2018; Bhattachan *et al.*, 2019; Tully *et al.*, 2019; Cao *et al.*, 2024). We introduced a 5 ppt salinity increase to induce a measurable physiological response and simulate saltwater intrusion, allowing us to assess the potential effects of contaminants interacting with a natural environmental stressor.

We observed that a sudden increase in salinity elevated NLR in all groups. However, the intensity of this response differed among the five groups. NLR increases were smaller in the PFPeA high group compared to the Cd low, Cd high, PFPeA low, and control groups. One possible explanation is that elevated pre-salinity NLR in the PFPeA high group limited the magnitude of subsequent leukocyte redistribution following salt exposure, resulting in a smaller relative change in NLR. Alternatively, PFPeA exposure may have altered physiological pathways associated with leukocyte dynamics, resulting in a different response pattern following the environmental perturbation. The PFPeA low, Cd-exposed, and control bass, which began at lower NLR levels, displayed more substantial—but temporary—NLR increases. Although the direct mechanisms underlying our observed patterns remain unclear, our results suggest that contaminant exposure may influence the interpretation of leukocyte-based stress biomarkers, and potentially other stress biomarkers, when organisms experience new environmental challenges. Such contaminant-associated changes in physiological responses may have implications for resilience in natural populations exposed to chronic contamination.

### Post-salinity exposure (blood samples 3 vs 4)

Seven days after the environmental stressor was introduced, NLR values in all groups except PFPeA high declined toward baseline levels. In contrast, NLR remained elevated in the PFPeA high group throughout the study period. The persistent rise of NLR in the PFPeA high group suggests that PFPeA exposure may alter leukocyte dynamics associated with physiological stress responses in largemouth bass. PFPeA, along with other PFAS compounds, may influence an individual’s ability to acclimatize to additional environmental stressors. Our findings suggest that PFAS exposure may contribute to sustained physiological responses and disrupt physiological allostasis. Previous research supports this interpretation, as PFAS exposure has altered fish leukocyte counts (Guillette *et al.*, 2020) and affected other physiological processes, including liver morphology (Piva *et al.*, 2022) and thyroid function (Ask *et al.*, 2021; Birgersson *et al.*, 2021), across different species. Our study extends these findings by suggesting that PFPeA exposure may contribute to sustained NLR elevation following an additional environmental perturbation.

A decrease in NLR across the control, Cd, and PFPeA low groups suggests that a 5 ppt salinity increase may be an acute but recoverable perturbation for bass. This aligns with previous research showing that bass tolerate moderate salinity (5–10 ppt; Yi *et al.*, 2021) and persist at levels below 8.7 ppt (Viadero *et al.*, 2025). However, higher salinity regimes (~15 ppt) can be lethal for bass (Norris *et al.*, 2010). Interestingly, we observed consistent declines in NLR across the control, Cd, and PFPeA low groups, suggesting no evidence that Cd or low-level PFPeA exposure altered post-salinity NLR trajectories relative to controls. Our results suggest that a moderate salinity increase represents a temporary physiological perturbation for largemouth bass under controlled laboratory conditions, although contaminant exposure may influence leukocyte responses following salinity change.

### Potential mechanisms, limitations, and future directions

Although the direct mechanisms underlying our observations remain uncertain, several plausible pathways may explain the sustained NLR elevation observed in PFPeA-exposed bass. PFAS compounds have been linked to endocrine disruption, including altered thyroid and glucocorticoid signalling (Ask *et al.*, 2021; Birgersson *et al.*, 2021; Coperchini *et al.*, 2021), immune modulation (Fang *et al.*, 2013; Guillette *et al.*, 2020), and oxidative stress (Piva *et al.*, 2022). Because leukocyte redistribution is closely associated with endocrine and immune regulation, disruption of these pathways may contribute to altered NLR dynamics following PFPeA exposure. These mechanisms remain hypothetical in the context of our study, but provide biologically plausible explanations for our observed patterns.

NLRs are widely used physiological biomarkers associated with stress responses across vertebrate taxa (Davis *et al.*, 2008; Goessling *et al.*, 2015; Watson *et al.*, 2020). One strength of NLR is that leukocyte profiles often remain informative during prolonged perturbations, when glucocorticoid concentrations may fluctuate, attenuate, or become more difficult to interpret over time (Davis *et al.*, 2008; Goessling *et al.*, 2015). Nevertheless, NLR represents only one component of an organism’s physiological response. Additional biomarkers, including cortisol, oxidative stress indicators, and other immune parameters, would provide a more comprehensive understanding of the physiological pathways influenced by contaminant exposure and environmental change.

Several limitations should be considered when interpreting our findings. First, our study involved relatively few individuals per treatment group and should therefore be viewed as an exploratory laboratory investigation aimed at identifying potential physiological responses, warranting further study rather than a definitive assessment. Second, the high PFPeA and Cd concentrations exceeded levels currently reported in natural aquatic systems. We intentionally included elevated concentrations to evaluate physiological responses across an order-of-magnitude scale, but future studies should investigate using finer-scale dosing increments and concentrations that reflect environmentally relevant conditions in greater detail. Additional work incorporating larger sample sizes, longer exposure durations, multiple physiological endpoints (e.g. endocrine, immunological, and metabolic), and environmentally realistic contaminant concentrations would further improve our understanding of xenobiotic influences on physiological biomarkers and help clarify the mechanistic basis of these responses.

### Implications from xenobiotic–stress relationships

Our findings have broad implications for understanding how contaminants affect vertebrate physiological biomarkers and the health of aquatic organisms, with relevance to conservation and ecological research. Leukocyte profiles, particularly NLRs, are widely used physiological biomarkers associated with stress responses across vertebrate taxa due to the conservative nature of the stress-related leukocyte response (Davis *et al.*, 2008). Therefore, it is reasonable to suggest that PFPeA, and potentially other xenobiotics, including short-chain PFAS isomers, may alter leukocyte dynamics associated with physiological stress responses in various species.

Studies on mammals have reported that certain PFAS compounds may affect lymphocyte and neutrophil activity differently (Qazi *et al.*, 2009), suggesting that contaminant-associated changes in leukocyte profiles may occur across diverse taxa. Combined with our findings, this evidence indicates that contaminant exposure may extend beyond endocrine disruption to include immune variation.

In the context of conservation physiology, our results highlight an important but often overlooked challenge: contaminant exposure may influence the interpretation of physiological biomarkers used to assess environmental disturbance. Natural populations are increasingly exposed to complex cocktails of exogenous pollutants (Kurwadkar *et al.*, 2022), yet physiological biomarkers are frequently interpreted without considering potential contaminant-associated effects. If xenobiotic exposure alters biomarker responses independently of the environmental perturbations under investigation, researchers and managers may draw inaccurate conclusions regarding organismal condition, environmental stress, or ecosystem health. Clarifying the mechanistic connections underlying xenobiotic interactions with organisms is essential for interpreting physiological indicators and strengthening conservation and management decisions in increasingly altered ecosystems.

## Data Availability

The data and code supporting this study’s findings, following the standards set out by Jenkins *et al.* (2023), are openly available on GitHub: https://github.com/tscoleman3/bass_stress_pfas_cd_aquaculture.
